# Poly[4,4′-imino­dipyridinium [tetra-μ_3_-oxido-tetra­oxido-di-μ_4_-phosphato-κ^4^ *O*:*O*′:*O*′′:*O*′′′-tetra­vanadium(V)]]

**DOI:** 10.1107/S1600536808038348

**Published:** 2008-11-22

**Authors:** Gregory A. Farnum, Robert L. LaDuca

**Affiliations:** a Lyman Briggs College, Department of Chemistry, Michigan State University, East Lansing, MI 48825, USA

## Abstract

In the title salt, {(C_10_H_11_N_3_)[V_4_O_8_(PO_4_)_2_]}_*n*_, cubane-like [V_4_O_8_]^4+^ clusters are connected by phosphate anions into anionic [V_4_P_2_O_16_]_*n*_^2*n*−^ layers. These aggregate into the three-dimensional structure *via* N—H⋯O hydrogen-bonding mechanisms imparted by 4,4′-imino­dipyridinium dications situated between the layers.

## Related literature

For a nickel vanadate phase incorporating 4,4′-dipyridylamine, see: LaDuca *et al.* (2001 ▶). For a related layered vanadium phosphate solid containing doubly protonated 4,4′-bipyridine cations, see: Shi *et al.* (2004 ▶).
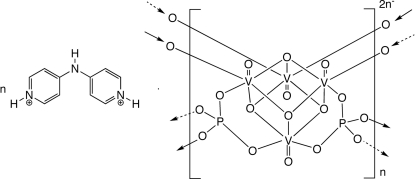


## Experimental

### 

#### Crystal data

(C_10_H_11_N_3_)[V_4_O_8_(PO_4_)_2_]
*M* *_r_* = 694.92
Monoclinic, 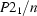

*a* = 7.4431 (10) Å
*b* = 14.524 (2) Å
*c* = 18.825 (3) Å
β = 94.363 (2)°
*V* = 2029.1 (5) Å^3^
*Z* = 4
Mo *K*α radiation
μ = 2.04 mm^−1^
*T* = 173 (2) K
0.20 × 0.20 × 0.04 mm

#### Data collection

Bruker SMART 1K diffractometer
Absorption correction: multi-scan (*SADABS*; Sheldrick, 1996 ▶) *T*_min_ = 0.786, *T*_max_ = 0.922
21687 measured reflections
4652 independent reflections
3678 reflections with *I* > 2σ(*I*)
*R*_int_ = 0.047

#### Refinement

*R*[*F* ^2^ > 2σ(*F* ^2^)] = 0.038
*wR*(*F* ^2^) = 0.101
*S* = 1.09
4652 reflections
325 parameters
3 restraints
H atoms treated by a mixture of independent and constrained refinement
Δρ_max_ = 1.03 e Å^−3^
Δρ_min_ = −0.76 e Å^−3^

### 

Data collection: *SMART* (Bruker, 2003 ▶); cell refinement: *SAINT* (Bruker, 2003 ▶); data reduction: *SAINT*; program(s) used to solve structure: *SHELXS97* (Sheldrick, 2008 ▶); program(s) used to refine structure: *SHELXL97* (Sheldrick, 2008 ▶); molecular graphics: *CrystalMaker* (Palmer, 2007 ▶); software used to prepare material for publication: *SHELXL97*.

## Supplementary Material

Crystal structure: contains datablocks I, global. DOI: 10.1107/S1600536808038348/tk2326sup1.cif

Structure factors: contains datablocks I. DOI: 10.1107/S1600536808038348/tk2326Isup2.hkl

Additional supplementary materials:  crystallographic information; 3D view; checkCIF report

## Figures and Tables

**Table 1:** Hydrogen-bond geometry (Å, °)

*D*—H⋯*A*	*D*—H	H⋯*A*	*D*⋯*A*	*D*—H⋯*A*
N1—H1N⋯O10	0.93 (4)	2.14 (4)	2.885 (4)	136 (4)
N1—H1N⋯O9^i^	0.93 (4)	2.45 (4)	3.018 (5)	119 (4)
N2—H2N⋯O2^ii^	0.862 (19)	2.35 (2)	3.195 (4)	166 (4)
N3—H3N⋯O8^iii^	0.90 (5)	2.02 (5)	2.902 (4)	164 (4)

Symmetry codes: (i) 
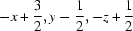
; (ii) 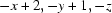; (iii) 
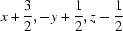
.

**Fig. 1.**
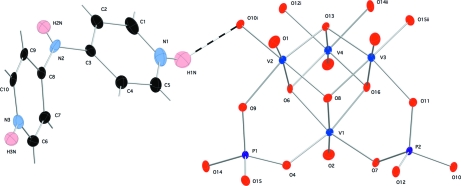
Asymmetric unit of (I), showing 50% probability ellipsoids and atom numbering scheme. Most H atom positions are shown as gray sticks. Color code: dark blue V, violet P, light blue N, red O, black C, pink H.

**Fig. 2.**
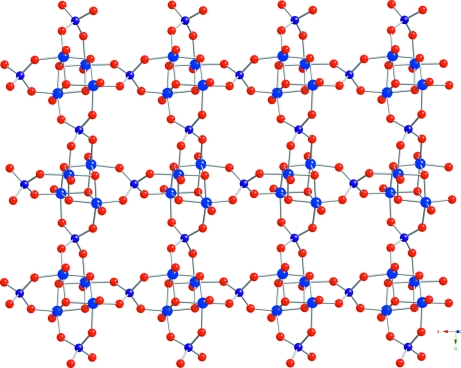
A single [V4P2O16]n2n- layer in (I).

**Fig. 3.**
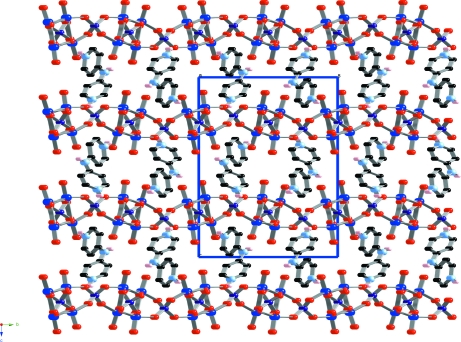
Packing diagram illustrating the ABAB layer stacking pattern, which forms the 3-D crystal structure of (I) through hydrogen bonding between the inorganic layers and 4,4'-iminodipyridinium cations.

## supplementary crystallographic information

### Comment

The kinked and hydrogen-bonding capable imine 4,4'-dipyridylamine has proven useful in the construction of novel mixed metal oxide phases (LaDuca *et al.*, 2001). In an attempt to extend this chemistry into a metal phosphate oxide system, yellow plate-like crystals of the title compound (I) were obtained.

The asymmetric unit of (I) comprises a cluster of four pentavalent V atoms, four terminal O atoms, four triply bridging O atoms, two phosphate anions and an unligated 4,4'-iminodipyridinium dication (Fig. 1). Each V atom is octahedrally coordinated, with three µ_3_O atom donors, two O atoms from two different phosphate anions, and one terminal O atom with a formal V=O double bond. The four V=O groups and four µ_3_ O atoms form a cubane-type [V_4_O_8_]^4+^ cluster.

Quadruply bridging phosphate anions bridge these cationic clusters into anionic [V_4_O_8_(PO_4_)_2_]_n_^2n-^ layers that are situated parallel to the *bc*-planes (Fig. 2). The phosphate groups bracket rhomboid apertures within the layers, with through-space P···P contact distances of 7.2685 (2) and 7.4431 (2) Å. Adjacent [V_4_O_8_(PO_4_)_2_]_n_^2n-^ layers stack in an *AB* pattern into the 3-D structure by N—H···O hydrogen bonding mediated by the protonated pyridyl-N atoms and the central amine groups of the 4,4'-iminodipyridinium cations situated in the interlamellar regions (Fig. 3).

The overall structure of (I) is very similar to a related phase incorporating doubly protonated 4,4'-bipyridine cations (Shi *et al.*, 2004).

### Experimental

All chemicals were obtained commercially. Vanadium(V) oxide (140 mg, 0.77 mmol) and 4,4'-dipyridylamine (132 mg, 0.77 mmol) and phosphoric acid (526 mg of an 85.5% aqueous solution, 4.56 mmol) were placed into H_2_O (10 ml ) in a 23 ml Teflon-lined Parr acid digestion bomb. The bomb was heated at 393 K for 72 h and was then allowed to cool to room temperature. Yellow plates of (I) were obtained along with a reddish-brown amorphous solid.

### Refinement

All H atoms bound to C atoms were placed in calculated positions with C—H = 0.95 Å and refined in riding mode with *U*_iso_ = 1.2*U*_eq_(C). All H atoms bound to N atoms were found *via* Fourier difference map, restrained with N—H = 0.89 Å, and refined with *U*_iso_=1.2*U*_eq_(N). The largest residual electron density peak of 1.03 e^-^ Å^-3^ was located 2.25 Å from the H2 atom.

### Crystal data

**Table:**

(C_10_H_11_N_3_)[V_4_O_8_(PO_4_)_2_]	*F*_000_ = 1368
*M**_r_* = 694.92	*D*_x_ = 2.275 Mg m^−^^3^
Monoclinic, *P*2_1_/*n*	Mo *K*α radiation λ = 0.71073 Å
Hall symbol: -P 2yn	Cell parameters from 21687 reflections
*a* = 7.4431 (10) Å	θ = 1.8–28.2º
*b* = 14.524 (2) Å	µ = 2.04 mm^−^^1^
*c* = 18.825 (3) Å	*T* = 173 (2) K
β = 94.363 (2)º	Plate, yellow
*V* = 2029.1 (5) Å^3^	0.20 × 0.20 × 0.04 mm
*Z* = 4	

### Data collection

**Table:**

Bruker SMART 1K diffractometer	4652 independent reflections
Radiation source: fine-focus sealed tube	3678 reflections with *I* > 2σ(*I*)
Monochromator: graphite	*R*_int_ = 0.047
*T* = 173(2) K	θ_max_ = 28.2º
ω scans	θ_min_ = 1.8º
Absorption correction: multi-scan(SADABS; Sheldrick, 1996)	*h* = −9→9
*T*_min_ = 0.786, *T*_max_ = 0.922	*k* = −19→19
21687 measured reflections	*l* = −24→24

### Refinement

**Table:**

Refinement on *F*^2^	Secondary atom site location: difference Fourier map
Least-squares matrix: full	Hydrogen site location: inferred from neighbouring sites
*R*[*F*^2^ > 2σ(*F*^2^)] = 0.038	H atoms treated by a mixture of independent and constrained refinement
*wR*(*F*^2^) = 0.101	*w* = 1/[σ^2^(*F*_o_^2^) + (0.0382*P*)^2^ + 6.7132*P*] where *P* = (*F*_o_^2^ + 2*F*_c_^2^)/3
*S* = 1.09	(Δ/σ)_max_ = 0.001
4652 reflections	Δρ_max_ = 1.03 e Å^−^^3^
325 parameters	Δρ_min_ = −0.76 e Å^−^^3^
3 restraints	Extinction correction: none
Primary atom site location: structure-invariant direct methods	

### Special details

**Table:**

Geometry. All e.s.d.'s (except the e.s.d. in the dihedral angle between two l.s. planes) are estimated using the full covariance matrix. The cell e.s.d.'s are taken into account individually in the estimation of e.s.d.'s in distances, angles and torsion angles; correlations between e.s.d.'s in cell parameters are only used when they are defined by crystal symmetry. An approximate (isotropic) treatment of cell e.s.d.'s is used for estimating e.s.d.'s involving l.s. planes.
Refinement. Refinement of *F*^2^ against ALL reflections. The weighted *R*-factor *wR* and goodness of fit *S* are based on *F*^2^, conventional *R*-factors *R* are based on *F*, with *F* set to zero for negative *F*^2^. The threshold expression of *F*^2^ > σ(*F*^2^) is used only for calculating *R*-factors(gt) *etc*. and is not relevant to the choice of reflections for refinement. *R*-factors based on *F*^2^ are statistically about twice as large as those based on *F*, and *R*- factors based on ALL data will be even larger.

### Fractional atomic coordinates and isotropic or equivalent isotropic displacement parameters (Å^2^)

**Table:**

	*x*	*y*	*z*	*U*_iso_*/*U*_eq_	
V1	0.62200 (8)	0.45521 (4)	0.16868 (3)	0.00774 (14)	
V2	0.65671 (8)	0.54528 (4)	0.33252 (3)	0.00778 (14)	
V3	0.91535 (8)	0.41272 (4)	0.30933 (3)	0.00822 (14)	
V4	0.89067 (8)	0.58608 (4)	0.19276 (3)	0.00808 (14)	
P1	0.27074 (12)	0.50073 (6)	0.25058 (5)	0.00875 (19)	
P2	0.74459 (12)	0.25121 (6)	0.21337 (5)	0.00750 (19)	
O1	0.6496 (4)	0.53003 (17)	0.41627 (14)	0.0146 (6)	
O2	0.5880 (3)	0.47775 (17)	0.08568 (14)	0.0141 (6)	
O3	0.9438 (4)	0.38002 (18)	0.38988 (14)	0.0157 (6)	
O4	0.3769 (3)	0.45212 (17)	0.19477 (14)	0.0117 (5)	
O5	0.8869 (3)	0.62016 (17)	0.11231 (14)	0.0144 (5)	
O6	0.6464 (3)	0.57326 (16)	0.20696 (13)	0.0093 (5)	
O7	0.6413 (3)	0.32294 (16)	0.16610 (13)	0.0095 (5)	
O8	0.6626 (3)	0.42384 (16)	0.29886 (13)	0.0093 (5)	
O9	0.3989 (3)	0.54901 (17)	0.30680 (14)	0.0115 (5)	
O10	0.8333 (3)	0.18063 (16)	0.16577 (13)	0.0093 (5)	
O11	0.8977 (3)	0.29677 (16)	0.26015 (14)	0.0101 (5)	
O12	0.6105 (3)	0.20382 (16)	0.25905 (14)	0.0104 (5)	
O13	0.8962 (3)	0.54013 (16)	0.31834 (14)	0.0095 (5)	
O14	0.1453 (3)	0.57446 (16)	0.21577 (14)	0.0114 (5)	
O15	0.1609 (3)	0.42794 (16)	0.28734 (14)	0.0121 (5)	
O16	0.8712 (3)	0.46004 (16)	0.18407 (13)	0.0096 (5)	
N1	1.1851 (5)	0.2291 (3)	0.1230 (2)	0.0247 (8)	
H1N	1.113 (5)	0.212 (3)	0.1588 (19)	0.030*	
N2	1.5138 (4)	0.3207 (2)	−0.02781 (18)	0.0157 (7)	
H2N	1.481 (6)	0.370 (2)	−0.050 (2)	0.019*	
N3	1.9274 (4)	0.1840 (2)	−0.11738 (19)	0.0161 (7)	
H3N	2.016 (6)	0.152 (3)	−0.136 (2)	0.019*	
C1	1.1673 (5)	0.3154 (3)	0.0975 (2)	0.0215 (9)	
H1	1.0813	0.3544	0.1146	0.026*	
C2	1.2741 (5)	0.3461 (3)	0.0469 (2)	0.0174 (8)	
H2	1.2593	0.4054	0.0287	0.021*	
C3	1.4072 (5)	0.2878 (3)	0.0223 (2)	0.0128 (7)	
C4	1.4197 (5)	0.1975 (3)	0.0485 (2)	0.0180 (8)	
H4	1.5027	0.1566	0.0317	0.022*	
C5	1.3089 (6)	0.1704 (3)	0.0990 (2)	0.0223 (9)	
H5	1.3182	0.1110	0.1172	0.027*	
C6	1.9152 (5)	0.1759 (3)	−0.0472 (2)	0.0183 (8)	
H6	1.9969	0.1389	−0.0205	0.022*	
C7	1.7833 (5)	0.2217 (3)	−0.0142 (2)	0.0159 (8)	
H7	1.7759	0.2167	0.0347	0.019*	
C8	1.6598 (5)	0.2760 (2)	−0.0557 (2)	0.0133 (7)	
C9	1.6813 (5)	0.2865 (2)	−0.1276 (2)	0.0128 (7)	
H9	1.6058	0.3257	−0.1553	0.015*	
C10	1.8149 (5)	0.2386 (2)	−0.1577 (2)	0.0142 (8)	
H10	1.8279	0.2440	−0.2063	0.017*	

### Atomic displacement parameters (Å^2^)

**Table:**

	*U*^11^	*U*^22^	*U*^33^	*U*^12^	*U*^13^	*U*^23^
V1	0.0072 (3)	0.0038 (3)	0.0120 (3)	0.0000 (2)	−0.0006 (2)	−0.0003 (2)
V2	0.0073 (3)	0.0036 (3)	0.0125 (3)	−0.0003 (2)	0.0011 (2)	−0.0002 (2)
V3	0.0071 (3)	0.0039 (3)	0.0134 (3)	0.0005 (2)	−0.0003 (2)	0.0008 (2)
V4	0.0074 (3)	0.0041 (3)	0.0128 (3)	−0.0002 (2)	0.0014 (2)	0.0002 (2)
P1	0.0057 (4)	0.0041 (4)	0.0165 (5)	0.0002 (3)	0.0008 (3)	−0.0012 (3)
P2	0.0070 (4)	0.0027 (4)	0.0130 (4)	0.0005 (3)	0.0012 (3)	−0.0002 (3)
O1	0.0196 (14)	0.0111 (13)	0.0135 (14)	−0.0002 (10)	0.0040 (11)	−0.0003 (10)
O2	0.0155 (13)	0.0113 (13)	0.0151 (14)	0.0003 (10)	−0.0013 (10)	0.0011 (10)
O3	0.0179 (14)	0.0113 (13)	0.0174 (15)	0.0027 (10)	−0.0014 (11)	0.0018 (10)
O4	0.0079 (12)	0.0092 (12)	0.0181 (14)	−0.0003 (10)	0.0007 (10)	−0.0043 (10)
O5	0.0172 (13)	0.0104 (13)	0.0160 (14)	−0.0013 (10)	0.0033 (11)	0.0007 (10)
O6	0.0069 (11)	0.0049 (11)	0.0159 (13)	0.0026 (9)	0.0003 (9)	−0.0015 (9)
O7	0.0102 (12)	0.0027 (11)	0.0155 (14)	0.0013 (9)	−0.0006 (10)	−0.0007 (9)
O8	0.0090 (12)	0.0044 (11)	0.0147 (13)	−0.0019 (9)	0.0021 (10)	−0.0006 (9)
O9	0.0069 (12)	0.0094 (12)	0.0183 (14)	−0.0010 (9)	0.0019 (10)	−0.0049 (10)
O10	0.0094 (12)	0.0039 (11)	0.0148 (14)	0.0007 (9)	0.0020 (10)	0.0005 (9)
O11	0.0084 (11)	0.0036 (11)	0.0180 (14)	0.0005 (9)	−0.0012 (10)	0.0002 (10)
O12	0.0095 (12)	0.0052 (11)	0.0171 (14)	0.0000 (9)	0.0046 (10)	0.0012 (10)
O13	0.0081 (12)	0.0030 (11)	0.0175 (14)	−0.0002 (9)	0.0005 (10)	−0.0013 (10)
O14	0.0076 (11)	0.0043 (11)	0.0229 (15)	−0.0003 (9)	0.0050 (10)	0.0035 (10)
O15	0.0080 (12)	0.0054 (12)	0.0227 (15)	0.0000 (9)	0.0004 (10)	0.0023 (10)
O16	0.0090 (12)	0.0043 (11)	0.0157 (13)	0.0016 (9)	0.0014 (10)	−0.0007 (10)
N1	0.0218 (19)	0.033 (2)	0.0203 (19)	−0.0092 (16)	0.0068 (15)	−0.0002 (16)
N2	0.0182 (16)	0.0098 (15)	0.0197 (18)	0.0021 (13)	0.0064 (13)	0.0039 (13)
N3	0.0143 (16)	0.0121 (16)	0.0224 (19)	−0.0020 (13)	0.0040 (14)	−0.0034 (13)
C1	0.0154 (19)	0.029 (2)	0.021 (2)	−0.0051 (17)	0.0042 (16)	−0.0103 (17)
C2	0.0147 (19)	0.017 (2)	0.021 (2)	−0.0027 (15)	0.0012 (15)	−0.0053 (16)
C3	0.0111 (17)	0.0165 (18)	0.0107 (18)	−0.0032 (15)	0.0006 (13)	−0.0017 (14)
C4	0.0168 (19)	0.018 (2)	0.019 (2)	−0.0013 (15)	0.0033 (16)	0.0012 (16)
C5	0.021 (2)	0.023 (2)	0.023 (2)	−0.0058 (17)	0.0041 (17)	0.0069 (17)
C6	0.0160 (19)	0.0130 (19)	0.025 (2)	−0.0007 (15)	−0.0037 (16)	0.0032 (16)
C7	0.0170 (19)	0.0152 (19)	0.015 (2)	−0.0017 (15)	0.0013 (15)	0.0018 (15)
C8	0.0133 (17)	0.0080 (17)	0.018 (2)	−0.0048 (14)	−0.0003 (15)	−0.0016 (14)
C9	0.0157 (18)	0.0069 (16)	0.0155 (19)	−0.0057 (14)	0.0001 (14)	0.0002 (14)
C10	0.0185 (19)	0.0073 (17)	0.0171 (19)	−0.0077 (14)	0.0030 (15)	−0.0013 (14)

### Geometric parameters (Å, °)

**Table:**

V1—O2	1.598 (3)	P2—O11	1.536 (2)
V1—O16	1.857 (2)	P2—O7	1.538 (2)
V1—O6	1.863 (2)	P2—O10	1.543 (3)
V1—O4	1.925 (3)	N1—C1	1.345 (6)
V1—O7	1.927 (2)	N1—C5	1.357 (6)
V1—O8	2.488 (3)	N1—H1N	0.93 (4)
V2—O1	1.597 (3)	N2—C3	1.364 (5)
V2—O13	1.824 (2)	N2—C8	1.401 (5)
V2—O8	1.876 (2)	N2—H2N	0.862 (19)
V2—O9	1.944 (2)	N3—C6	1.336 (5)
V2—O10^i^	1.967 (2)	N3—C10	1.346 (5)
V2—O6	2.394 (3)	N3—H3N	0.90 (5)
V3—O3	1.588 (3)	C1—C2	1.362 (6)
V3—O13	1.865 (2)	C1—H1	0.9300
V3—O8	1.883 (2)	C2—C3	1.408 (5)
V3—O15^ii^	1.917 (3)	C2—H2	0.9300
V3—O11	1.921 (2)	C3—C4	1.402 (5)
V3—O16	2.454 (3)	C4—C5	1.363 (6)
V4—O5	1.592 (3)	C4—H4	0.9300
V4—O16	1.843 (2)	C5—H5	0.9300
V4—O6	1.867 (2)	C6—C7	1.373 (6)
V4—O14^ii^	1.919 (2)	C6—H6	0.9300
V4—O12^i^	1.936 (2)	C7—C8	1.404 (5)
V4—O13	2.454 (3)	C7—H7	0.9300
P1—O15	1.533 (3)	C8—C9	1.384 (5)
P1—O4	1.534 (3)	C9—C10	1.371 (5)
P1—O14	1.535 (3)	C9—H9	0.9300
P1—O9	1.539 (3)	C10—H10	0.9300
P2—O12	1.529 (3)		
			
O2—V1—O16	103.13 (13)	O12—P2—O11	111.02 (15)
O2—V1—O6	101.14 (12)	O12—P2—O7	108.15 (14)
O16—V1—O6	80.68 (10)	O11—P2—O7	110.88 (13)
O2—V1—O4	99.88 (13)	O12—P2—O10	110.73 (14)
O16—V1—O4	156.29 (11)	O11—P2—O10	106.72 (14)
O6—V1—O4	89.38 (11)	O7—P2—O10	109.35 (14)
O2—V1—O7	100.73 (12)	P1—O4—V1	135.36 (15)
O16—V1—O7	88.06 (10)	V1—O6—V4	95.95 (11)
O6—V1—O7	157.17 (11)	V1—O6—V2	102.74 (11)
O4—V1—O7	93.27 (11)	V4—O6—V2	101.53 (10)
O2—V1—O8	177.48 (11)	P2—O7—V1	134.08 (15)
O16—V1—O8	78.94 (10)	V2—O8—V3	95.40 (11)
O6—V1—O8	77.68 (10)	V2—O8—V1	98.99 (10)
O4—V1—O8	77.93 (10)	V3—O8—V1	99.46 (10)
O7—V1—O8	80.71 (9)	P1—O9—V2	135.01 (16)
O1—V2—O13	104.17 (13)	P2—O10—V2^iii^	132.20 (15)
O1—V2—O8	101.92 (12)	P2—O11—V3	132.42 (15)
O13—V2—O8	82.17 (10)	P2—O12—V4^iii^	133.42 (15)
O1—V2—O9	98.26 (13)	V2—O13—V3	97.83 (11)
O13—V2—O9	157.22 (12)	V2—O13—V4	100.65 (10)
O8—V2—O9	89.43 (10)	V3—O13—V4	100.26 (11)
O1—V2—O10^i^	97.26 (12)	P1—O14—V4^iv^	135.73 (15)
O13—V2—O10^i^	90.42 (10)	P1—O15—V3^iv^	136.61 (15)
O8—V2—O10^i^	160.60 (11)	V4—O16—V1	97.02 (11)
O9—V2—O10^i^	90.66 (10)	V4—O16—V3	100.90 (11)
O1—V2—O6	175.87 (11)	V1—O16—V3	101.46 (11)
O13—V2—O6	79.67 (10)	C1—N1—C5	121.2 (4)
O8—V2—O6	79.97 (10)	C1—N1—H1N	118 (3)
O9—V2—O6	78.02 (10)	C5—N1—H1N	121 (3)
O10^i^—V2—O6	81.07 (9)	C3—N2—C8	127.3 (3)
O3—V3—O13	102.45 (13)	C3—N2—H2N	118 (3)
O3—V3—O8	100.68 (13)	C8—N2—H2N	114 (3)
O13—V3—O8	80.90 (10)	C6—N3—C10	121.6 (3)
O3—V3—O15^ii^	100.40 (13)	C6—N3—H3N	117 (3)
O13—V3—O15^ii^	89.19 (11)	C10—N3—H3N	122 (3)
O8—V3—O15^ii^	158.20 (11)	N1—C1—C2	120.5 (4)
O3—V3—O11	101.38 (12)	N1—C1—H1	119.7
O13—V3—O11	155.60 (11)	C2—C1—H1	119.7
O8—V3—O11	89.63 (10)	C1—C2—C3	119.7 (4)
O15^ii^—V3—O11	91.63 (11)	C1—C2—H2	120.1
O3—V3—O16	178.86 (12)	C3—C2—H2	120.1
O13—V3—O16	78.69 (10)	N2—C3—C4	123.0 (3)
O8—V3—O16	79.38 (10)	N2—C3—C2	118.5 (4)
O15^ii^—V3—O16	79.66 (10)	C4—C3—C2	118.4 (4)
O11—V3—O16	77.48 (10)	C5—C4—C3	119.2 (4)
O5—V4—O16	103.23 (13)	C5—C4—H4	120.4
O5—V4—O6	102.74 (13)	C3—C4—H4	120.4
O16—V4—O6	80.95 (10)	N1—C5—C4	120.8 (4)
O5—V4—O14^ii^	100.81 (13)	N1—C5—H5	119.6
O16—V4—O14^ii^	90.13 (10)	C4—C5—H5	119.6
O6—V4—O14^ii^	156.16 (12)	N3—C6—C7	120.6 (4)
O5—V4—O12^i^	99.84 (12)	N3—C6—H6	119.7
O16—V4—O12^i^	156.34 (11)	C7—C6—H6	119.7
O6—V4—O12^i^	88.95 (10)	C6—C7—C8	118.8 (4)
O14^ii^—V4—O12^i^	90.67 (11)	C6—C7—H7	120.6
O5—V4—O13	177.67 (11)	C8—C7—H7	120.6
O16—V4—O13	79.09 (10)	C9—C8—N2	117.8 (3)
O6—V4—O13	77.30 (10)	C9—C8—C7	119.1 (4)
O14^ii^—V4—O13	79.32 (10)	N2—C8—C7	123.1 (4)
O12^i^—V4—O13	77.83 (10)	C10—C9—C8	119.3 (4)
O15—P1—O4	108.18 (14)	C10—C9—H9	120.3
O15—P1—O14	110.24 (14)	C8—C9—H9	120.3
O4—P1—O14	110.92 (15)	N3—C10—C9	120.4 (4)
O15—P1—O9	109.13 (15)	N3—C10—H10	119.8
O4—P1—O9	110.85 (14)	C9—C10—H10	119.8
O14—P1—O9	107.51 (14)		
			
O15—P1—O4—V1	−133.2 (2)	O8—V3—O11—P2	15.9 (2)
O14—P1—O4—V1	105.7 (2)	O15^ii^—V3—O11—P2	−142.4 (2)
O2—V1—O4—P1	−125.5 (2)	O16—V3—O11—P2	−63.3 (2)
O16—V1—O4—P1	40.4 (4)	O11—P2—O12—V4^iii^	−81.1 (2)
O6—V1—O4—P1	−24.3 (2)	O7—P2—O12—V4^iii^	157.06 (19)
O7—V1—O4—P1	133.0 (2)	O10—P2—O12—V4^iii^	37.3 (3)
O8—V1—O4—P1	53.2 (2)	O1—V2—O13—V3	85.66 (14)
O2—V1—O6—V4	−84.30 (13)	O8—V2—O13—V3	−14.73 (11)
O16—V1—O6—V4	17.36 (11)	O9—V2—O13—V3	−84.0 (3)
O4—V1—O6—V4	175.75 (12)	O10^i^—V2—O13—V3	−176.74 (12)
O7—V1—O6—V4	78.8 (3)	O6—V2—O13—V3	−95.89 (11)
O8—V1—O6—V4	97.97 (11)	O1—V2—O13—V4	−172.29 (11)
O2—V1—O6—V2	172.42 (11)	O8—V2—O13—V4	87.33 (11)
O16—V1—O6—V2	−85.91 (11)	O9—V2—O13—V4	18.0 (3)
O4—V1—O6—V2	72.48 (11)	O10^i^—V2—O13—V4	−74.69 (10)
O7—V1—O6—V2	−24.5 (3)	O6—V2—O13—V4	6.16 (8)
O8—V1—O6—V2	−5.31 (8)	O3—V3—O13—V2	−84.30 (14)
O5—V4—O6—V1	84.15 (13)	O8—V3—O13—V2	14.72 (11)
O16—V4—O6—V1	−17.49 (11)	O15^ii^—V3—O13—V2	175.22 (12)
O14^ii^—V4—O6—V1	−86.7 (3)	O11—V3—O13—V2	83.1 (3)
O12^i^—V4—O6—V1	−176.00 (12)	O16—V3—O13—V2	95.61 (11)
O13—V4—O6—V1	−98.25 (11)	O3—V3—O13—V4	173.31 (11)
O5—V4—O6—V2	−171.52 (11)	O8—V3—O13—V4	−87.67 (10)
O16—V4—O6—V2	86.85 (11)	O15^ii^—V3—O13—V4	72.83 (11)
O14^ii^—V4—O6—V2	17.6 (3)	O11—V3—O13—V4	−19.3 (3)
O12^i^—V4—O6—V2	−71.67 (11)	O16—V3—O13—V4	−6.78 (8)
O13—V4—O6—V2	6.09 (8)	O16—V4—O13—V2	−91.04 (12)
O1—V2—O6—V1	−110.6 (16)	O6—V4—O13—V2	−7.98 (11)
O13—V2—O6—V1	90.76 (11)	O14^ii^—V4—O13—V2	176.74 (12)
O8—V2—O6—V1	6.99 (11)	O12^i^—V4—O13—V2	83.71 (11)
O9—V2—O6—V1	−84.57 (11)	O16—V4—O13—V3	9.03 (11)
O10^i^—V2—O6—V1	−177.17 (12)	O6—V4—O13—V3	92.09 (11)
O13—V2—O6—V4	−8.13 (11)	O14^ii^—V4—O13—V3	−83.19 (11)
O8—V2—O6—V4	−91.91 (11)	O12^i^—V4—O13—V3	−176.22 (12)
O9—V2—O6—V4	176.53 (12)	O15—P1—O14—V4^iv^	−13.6 (3)
O10^i^—V2—O6—V4	83.94 (11)	O4—P1—O14—V4^iv^	106.2 (2)
O12—P2—O7—V1	104.0 (2)	O9—P1—O14—V4^iv^	−132.5 (2)
O11—P2—O7—V1	−18.0 (3)	O4—P1—O15—V3^iv^	−129.8 (2)
O10—P2—O7—V1	−135.4 (2)	O14—P1—O15—V3^iv^	−8.3 (3)
O2—V1—O7—P2	149.9 (2)	O9—P1—O15—V3^iv^	109.6 (2)
O16—V1—O7—P2	46.9 (2)	O5—V4—O16—V1	−83.47 (14)
O6—V1—O7—P2	−13.3 (4)	O6—V4—O16—V1	17.59 (11)
O4—V1—O7—P2	−109.4 (2)	O14^ii^—V4—O16—V1	175.38 (12)
O8—V1—O7—P2	−32.2 (2)	O12^i^—V4—O16—V1	83.4 (3)
O1—V2—O8—V3	−88.41 (13)	O13—V4—O16—V1	96.29 (11)
O13—V2—O8—V3	14.51 (11)	O5—V4—O16—V3	173.38 (11)
O9—V2—O8—V3	173.26 (12)	O6—V4—O16—V3	−85.56 (11)
O10^i^—V2—O8—V3	82.9 (3)	O14^ii^—V4—O16—V3	72.23 (11)
O6—V2—O8—V3	95.33 (10)	O12^i^—V4—O16—V3	−19.7 (3)
O1—V2—O8—V1	171.10 (11)	O13—V4—O16—V3	−6.86 (8)
O13—V2—O8—V1	−85.99 (11)	O2—V1—O16—V4	81.73 (13)
O9—V2—O8—V1	72.77 (10)	O6—V1—O16—V4	−17.64 (11)
O10^i^—V2—O8—V1	−17.6 (4)	O4—V1—O16—V4	−84.0 (3)
O6—V2—O8—V1	−5.16 (8)	O7—V1—O16—V4	−177.70 (12)
O3—V3—O8—V2	86.84 (13)	O8—V1—O16—V4	−96.79 (11)
O13—V3—O8—V2	−14.23 (11)	O2—V1—O16—V3	−175.60 (11)
O15^ii^—V3—O8—V2	−78.2 (3)	O6—V1—O16—V3	85.03 (11)
O11—V3—O8—V2	−171.65 (11)	O4—V1—O16—V3	18.7 (3)
O16—V3—O8—V2	−94.32 (10)	O7—V1—O16—V3	−75.03 (11)
O3—V3—O8—V1	−173.09 (11)	O8—V1—O16—V3	5.88 (8)
O13—V3—O8—V1	85.84 (10)	O13—V3—O16—V4	9.06 (11)
O15^ii^—V3—O8—V1	21.9 (3)	O8—V3—O16—V4	91.79 (11)
O11—V3—O8—V1	−71.58 (10)	O15^ii^—V3—O16—V4	−82.19 (11)
O16—V3—O8—V1	5.75 (8)	O11—V3—O16—V4	−176.21 (12)
O16—V1—O8—V2	89.44 (11)	O13—V3—O16—V1	−90.50 (12)
O6—V1—O8—V2	6.69 (10)	O8—V3—O16—V1	−7.77 (11)
O4—V1—O8—V2	−85.33 (11)	O15^ii^—V3—O16—V1	178.25 (12)
O7—V1—O8—V2	179.27 (12)	O11—V3—O16—V1	84.23 (11)
O16—V1—O8—V3	−7.62 (10)	C5—N1—C1—C2	−0.2 (6)
O6—V1—O8—V3	−90.37 (11)	N1—C1—C2—C3	−1.5 (6)
O4—V1—O8—V3	177.60 (12)	C8—N2—C3—C4	−6.6 (6)
O7—V1—O8—V3	82.21 (11)	C8—N2—C3—C2	175.7 (4)
O15—P1—O9—V2	106.2 (2)	C1—C2—C3—N2	−179.2 (4)
O4—P1—O9—V2	−12.8 (3)	C1—C2—C3—C4	3.0 (5)
O14—P1—O9—V2	−134.2 (2)	N2—C3—C4—C5	179.5 (4)
O1—V2—O9—P1	−129.9 (2)	C2—C3—C4—C5	−2.8 (6)
O13—V2—O9—P1	40.0 (4)	C1—N1—C5—C4	0.3 (6)
O8—V2—O9—P1	−27.9 (2)	C3—C4—C5—N1	1.2 (6)
O10^i^—V2—O9—P1	132.7 (2)	C10—N3—C6—C7	1.8 (6)
O6—V2—O9—P1	51.9 (2)	N3—C6—C7—C8	0.9 (6)
O12—P2—O10—V2^iii^	−14.4 (2)	C3—N2—C8—C9	141.3 (4)
O11—P2—O10—V2^iii^	106.6 (2)	C3—N2—C8—C7	−38.6 (6)
O7—P2—O10—V2^iii^	−133.43 (19)	C6—C7—C8—C9	−4.0 (5)
O12—P2—O11—V3	−82.1 (2)	C6—C7—C8—N2	175.9 (3)
O7—P2—O11—V3	38.1 (3)	N2—C8—C9—C10	−175.4 (3)
O10—P2—O11—V3	157.15 (19)	C7—C8—C9—C10	4.5 (5)
O3—V3—O11—P2	116.7 (2)	C6—N3—C10—C9	−1.3 (5)
O13—V3—O11—P2	−50.7 (4)	C8—C9—C10—N3	−1.9 (5)

Symmetry codes: (i) −*x*+3/2, *y*+1/2, −*z*+1/2; (ii) *x*+1, *y*, *z*; (iii) −*x*+3/2, *y*−1/2, −*z*+1/2; (iv) *x*−1, *y*, *z*.

### Hydrogen-bond geometry (Å, °)

**Table:**

*D*—H···*A*	*D*—H	H···*A*	*D*···*A*	*D*—H···*A*
N1—H1N···O10	0.93 (4)	2.14 (4)	2.885 (4)	136 (4)
N1—H1N···O9^iii^	0.93 (4)	2.45 (4)	3.018 (5)	119 (4)
N2—H2N···O2^v^	0.862 (19)	2.35 (2)	3.195 (4)	166 (4)
N3—H3N···O8^vi^	0.90 (5)	2.02 (5)	2.902 (4)	164 (4)

Symmetry codes: (iii) −*x*+3/2, *y*−1/2, −*z*+1/2; (v) −*x*+2, −*y*+1, −*z*; (vi) *x*+3/2, −*y*+1/2, *z*−1/2.

## References

[bb1] Bruker (2003). *SMART* and *SAINT* Bruker AXS Inc., Madison, Wisconsin, USA.

[bb2] LaDuca, R. L., Rarig, R. S. & Zubieta, J. (2001). *Inorg. Chem.* **40**, 607–612. 10.1021/ic000224i 11225100

[bb3] Palmer, D. (2007). *CrystalMaker* CrystalMaker Software Ltd, Bicester, Oxfordshire, England.

[bb4] Sheldrick, G. M. (1996). *SADABS* University of Göttingen, Germany.

[bb5] Sheldrick, G. M. (2008). *Acta Cryst.* A**64**, 112–122. 10.1107/S0108767307043930 18156677

[bb6] Shi, F.-N., Paz, F. A. A., Rocha, J., Klinowski, Ja. & Trindade, T. (2004). *Eur. J. Inorg. Chem.* pp.3031–3037.

